# Coparenting and Parenting Pathways From the Couple Relationship to Children’s Behavior Problems

**DOI:** 10.1037/fam0000492

**Published:** 2018-12-27

**Authors:** Alison Parkes, Michael Green, Kirstin Mitchell

**Affiliations:** 1 MRC/CSO Social and Public Health Sciences Unit, College of Medical, Veterinary & Life Sciences, University of Glasgow

**Keywords:** couple relationship, parenting, coparenting, externalizing behavior problems

## Abstract

Although an extensive literature has linked couple conflict with the development of children’s externalizing behavior problems, longer term protective effects of positive dimensions of couple relationships on children’s externalizing behavior remain understudied, particularly in relation to underlying mechanisms. Supportiveness in the dyadic couple relationship may enhance mothers’ and fathers’ individual parenting skills and protect against children’s behavior problems, but the contribution of coparenting (couples’ support for one another’s individual parenting) remains unclear. This observational study investigated associations between couple supportiveness in children’s infancy and middle childhood externalizing problems, exploring pathways involving coparenting and/or mothers’ and fathers’ individual parenting using data from the U.K. Millennium Cohort Study (MCS; *N* = 5,779) and the U.S. Fragile Families and Child Wellbeing Study (FFS; *N* = 2,069). Couple supportiveness was associated with reduced externalizing problems 8 to 10 years later (standardized betas: MCS = –.13, FFS = −.11, both *p*s < .001). Much of this effect (60% MCS, 55% FFS) was attributable to coparenting and parenting when children were aged 3 to 5 years. Pathways from couple supportiveness involving negative parenting were stronger than those via positive parenting, pathways via mothers’ parenting were stronger than those via fathers’ parenting, and there were pathways via coparenting alone (without affecting parenting). Pathways involving coparenting were similar in magnitude (MCS), or larger (FFS), than those involving parenting alone. Consistent findings across different population samples suggest that helping parents to support one another in coparenting and to develop their individual parenting skills may lessen the longer term impact of couple relationship problems during early childhood.

Externalizing problems such as aggression, rule-breaking, and attentional problems often develop early in childhood (0 to 5 years) and then typically decline across middle childhood (6 to 12 years; Petersen, Bates, Dodge, Lansford, & Pettit, 2015), but persisting or increasing problems during middle childhood signal compromised psychosocial functioning across a wide range of adverse adult outcomes (Odgers et al., 2008). Early childhood is a key period of social and cognitive development that can protect against later externalizing problems (Fearon, Bakermans-Kranenburg, van Ijzendoorn, Lapsley, & Roisman, 2010; Schoemaker, Mulder, Deković, & Matthys, 2013), so it is critical to understand potentially modifiable aspects of family functioning that can impinge on this early development. Yet despite the theorized central importance of the couple relationship in supporting the key role of parenting during the child’s early years (Belsky, 1984), surprisingly little is known about how the couple relationship during early childhood affects children’s longer-term development (Pendry & Adam, 2013). Two meta-analyses dating from the 1990s established that negative aspects of couple relationship quality, especially conflict, constitute a small to moderate risk factor for children’s externalizing behavior (Buehler et al., 1997; Reid & Crisafulli, 1990). Conversely, positive aspects of the couple relationship such as satisfaction and supportiveness, while less well-studied than conflict, have protective effects (e.g., Goldberg & Carlson, 2014). Most existing longitudinal studies are, however, limited to relatively short follow-up periods, although a recent study found that interparental conflict in early childhood predicted higher levels of externalizing problems from age 5 to adulthood (Petersen et al., 2015).

More research is required to establish whether there are similar long term effects of positive dimensions of couple relationships, and to explore underlying mechanisms. This study explores associations between couple supportiveness in the child’s infancy and middle childhood externalizing behavior problems. It investigates possible pathways, via coparenting and parenting.

## Indirect Effects of Couple Relationship Quality via Parenting and Coparenting

The main theory indirectly linking the quality of the dyadic couple relationship with child socioemotional adjustment, the spillover hypothesis, suggests two main mechanisms involving disruptions to family processes concerning the child (Erel & Burman, 1995). First, tensions in the couple relationship may lead to compromised parent–child interactions (parenting), as parents become less emotionally sensitive to the child’s needs. This idea receives considerable empirical support, in relation to both couple conflict and lower marital satisfaction (Erel & Burman, 1995; Krishnakumar & Buehler, 2000). Second, couple relationship problems may undermine supportive coparenting. Coparenting concerns parents’ joint family management and division of labor, agreement on child rearing and support for one another’s parenting (Feinberg, 2003). Although often operationalised as a particular domain of dyadic couple functioning, a family systems approach classifies coparenting as triadic functioning, where the child is also involved (Talbot & McHale, 2004). Empirical work supports a distinction between triadic coparenting and dyadic couple relationship functioning, finding that some couples with a poor dyadic relationship may engage in supportive triadic coparenting, and vice versa (Feinberg, 2003). Nonetheless, positive qualities of the dyadic couple relationship are likely to facilitate supportive coparenting, which may in turn help sustain dyadic parent–child relationships (Fincham & Hall, 2005). Empirical support for the idea that coparenting is an intermediary on spillover paths from the dyadic couple relationship to mothers’ and fathers’ individual parenting comes from several studies of families with young children, (e.g., Holland & McElwain, 2013; Pedro, Ribeiro, & Shelton, 2012).

**Figure 1 fig1:**
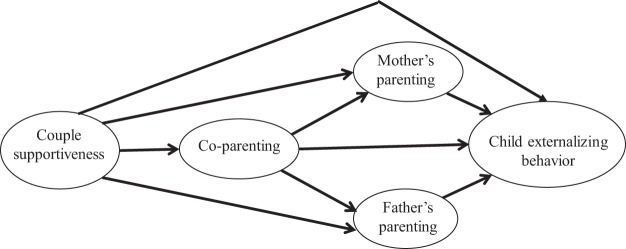
Conceptual model of pathways from couple relationship quality to children’s externalizing problems.

The conceptual model in Figure 1 extends the spillover model of couple relationship qualities onto coparenting and parenting (Erel & Burman, 1995; Fincham & Hall, 2005), to show their impact on children’s externalizing behavior. It suggests indirect paths from the couple relationship via parenting alone, via coparenting and then parenting, and via coparenting alone. The part of the model extending parenting spillover to impact externalizing problems is well-established, as many studies have linked less positive parenting (e.g., warmth) and more negative parenting (e.g., harsh control) with the development of externalizing problem behavior (Pinquart, 2017). The model also suggests a pathway involving coparenting alone, since there is empirical evidence for independent effects of coparenting and parenting on children’s externalizing behavior (Teubert & Pinquart, 2010). This pathway is less well understood than those involving parenting. Overt coparenting disagreements might model irritability, anger and aggression, disinhibiting the use of such behavior regardless of parents’ individual parenting effectiveness (Chen & Johnston, 2012). Even when the child is not directly exposed to coparenting tensions, lack of cooperation and/or undermining coparenting might model negative behavior strategies, such as noncompliance with rules or bullying (Murphy, Jacobvitz, & Hazen, 2016).[Fig-anchor fig1]

There is mounting empirical evidence for long-term indirect effects of the couple relationship in early childhood on child behavior, via enduring effects on parenting quality. Several longitudinal studies of families with young children have found indirect paths from the couple relationship via positive and negative parenting, acting over one or more years (Gerard, Krishnakumar, & Buehler, 2006; Lindsey, Caldera, & Tankersley, 2009; Rhoades et al., 2011; Schoppe-Sullivan, Schermerhorn, & Cummings, 2007; Stover et al., 2016). In contrast, research that explores coparenting as an additional mediator is relatively sparse: two studies (O’Leary & Vidair, 2005; Stroud, Meyers, Wilson, & Durbin, 2015) consider both coparenting and parenting as mediators, finding indirect pathways via both types of process to externalizing problems. However, only one study allowed for all the paths suggested by our conceptual model (O’Leary & Vidair, 2005), and neither used a longitudinal design.

In addition to a paucity of research on a more comprehensive set of indirect pathways, there are other notable research gaps left by existing pathway studies of families with young children. The studies cited above examining pathways from the couple relationship to children’s externalizing problems via parenting all used couple conflict or hostility as the main exposure, and there is a need to explore pathways to children’s problems from alternative, more positive dimensions of the couple relationship. There are also question marks over the generalizability of existing studies. All but one (Gerard et al., 2006) of the parenting pathway studies relied on small, convenience samples with little or no allowance for potential confounders. A further limitation of existing longitudinal pathway studies is that they do not generally span the critical period of the child’s transition to school, relating either to preschool (Lindsey et al., 2009; Rhoades et al., 2011) or to school-age years (Gerard et al., 2006; Schoppe-Sullivan et al., 2007). The one exception covering school transition found that effects of the couple relationship were confined to the preschool period (Stover et al., 2016). More studies are therefore required to establish whether indirect pathways extend from couple relationship quality in early childhood, to impact on school-age externalizing problems.

## Study Aims and Hypotheses

This is the first longitudinal study to assess the independent contribution of coparenting, as well as parenting, toward explaining the effects of couple relationship quality during children’s early years on middle childhood externalizing problems. We define relationship quality in terms of overall mutual emotional supportiveness, as this is likely to convey important positive as well as negative dimensions of couple functioning (Goldberg & Carlson, 2014). We assess the couple relationship in infancy as this is a good guide to subsequent supportiveness across the preschool years (Howard & Brooks-Gunn, 2009), and predates the development of behavior problems thereby excluding reverse causation (but not confounding). We assess potential coparenting and parenting mediators mainly in the preschool years (including some information from the school transition period in one of the study samples). Coparenting and parenting of preschoolers is important for the development of executive functioning and secure attachment (Fay-Stammbach, Hawes, & Meredith, 2014; Karreman, van Tuijl, van Aken, & Dekovic, 2008; Moss, Cyr, Bureau, Tarabulsy, & Dubois-Comtois, 2005; Pérez, Moessner, & Santelices, 2017), which in turn protect against externalizing problem development (Fearon et al., 2010; Schoemaker et al., 2013). Thus, we are able to study our potential mediators at an important point. Nonetheless, because coparenting and parenting behaviors are moderately stable in early childhood (Dallaire & Weinraub, 2005; Laxman et al., 2013), we are likely to also capture the quality of these processes during the child’s infancy and toddler years.

We explore the following hypotheses: (1) couple supportiveness will be associated with reduced childhood externalizing problems in middle childhood and (2) associations will be mediated by supportive coparenting and higher quality mother’s and father’s individual parenting of the preschool child. On the basis of previous literature, finding independent effects of parenting and coparenting on externalizing problems (Teubert & Pinquart, 2010), we expect additive indirect effects of coparenting and parenting constructs, although it is not clear whether coparenting (followed by parenting or alone) channels most of the spillover effect of the couple relationship (O’Leary & Vidair, 2005; Stroud et al., 2015). Previous pathway studies also lead us to expect indirect effects involving both negative and positive parenting (Gerard et al., 2006; Lindsey et al., 2009; Schoppe-Sullivan et al., 2007; Stover et al., 2016), but it is not clear where the balance of effects lies. Another unresolved issue is whether spillover has a more pronounced effect on one parent; either the mother as the child’s main caregiver, or the father, whose involvement with the child, as suggested by the fathering vulnerability hypothesis, may be relatively dependent on partner support (Cummings, Merrilees, & Ward George, 2010). Empirical studies addressing the question of whether spillover affects one parent more than the other have inconsistent findings (Lindsey et al., 2009; Rhoades et al., 2011; Stover et al., 2016; Stroud et al., 2015). In connection with our second hypothesis, we explore the following comparisons related to the magnitude of distinct indirect pathways to clarify which mechanisms are more dominant:

1 coparenting (with/without parenting) versus parenting alone,
2 negative parenting versus positive parenting, and
3 mother’s parenting versus father’s parenting.

We use two large longitudinal population-based samples, drawn from different countries, with different ethnic and sociodemographic compositions, so similar results will increase our confidence in the generalizability of the mechanisms. The two study samples have distinct, complementary characteristics. The first consists of a sample of stable couple families containing both biological parents coresiding throughout the child’s early years. As a nationally representative sample, the U.K. Millennium Cohort Study (MCS) offers more generalizability than existing studies using convenience samples. The second draws from a large population-based study from the United States, the Fragile Families and Child Wellbeing Study (FFS). This study was designed to oversample unmarried parents, who are underrepresented in research on couple relationship problems in relation to child adjustment. Unlike the U.K. study, the U.S. study included data on couple supportiveness, coparenting, and parenting from non-coresiding couples.

## Method

This study drew on data from two cohort studies. As a secondary data analysis, ethical approval was deemed unnecessary.

### Sample 1: MCS

The MCS is a prospective study of U.K. children born between September 2000 and January 2002. The stratified clustered sampling design ensures good representation of children from disadvantaged areas, ethnic minority groups and from all four U.K. countries (Plewis, Calderwood, Hawkes, Hughes, & Joshi, 2007). Families were first interviewed in-home at 9 months postbirth, when 18,552 families were contacted (96% of those eligible to take part). Interviews were repeated when children were aged 3, 5, 7, and 11, and teachers completed postal surveys at ages 7 and 11. We excluded families with respondents in Scotland and Northern Ireland (because teachers were surveyed only in England and Wales; *n* = 4,259), families in England and Wales with multiple births (*n* = 194), and those where the household did not contain two resident parents (as only coresident parents were interviewed; *n* = 2,471). Out of the remaining 11,628 couple families from England and Wales with a singleton child, 8,721 families were followed up at ages 3 and 5. Because full information on mediator measures was only collected from coresiding couples living together at these time points, we dropped families where couples had separated (*n* = 1,276), leaving 7,445 eligible families. The analytic sample was restricted to 5,779 of these, where information on externalizing problems at the final time point, age 11, was provided by at least one source (parent, teacher, child).

### Sample 2: FFS

The FFS is a longitudinal study of families with a child born between 1998 and 2000 in 20 large U.S. cities (>200,000 inhabitants; *N* = 4,897). It was designed to oversample unmarried couples: these represented three quarters of the sample at baseline, with 40% not living together when their child was born (Reichman, Teitler, Garfinkel, & McLanahan, 2001). Mothers and fathers were interviewed in the hospital within 48 hr of the child’s birth, and follow-up information was collected by telephone when the child was approximately 1, 3, 5, and 9 years old. In-home assessments were performed at 3, 5, and 9 years, and teachers completed postal questionnaires at 5 and 9 years. At child aged 1 year, there were 4,364 families where the mother was interviewed. We excluded families with multiple births (*n* = 83), families where the mother was not married or in a romantic relationship with the child’s father (*n* = 1,408), and those where neither parent provided information on couple relationship quality (*n* = 3), leaving 2,873 eligible families. The analytic sample was limited to 2,069 families where information was available on the 9-year-old child’s externalizing problems from at least one source (parent, teacher, child).

### Measures

Here, we outline the main measures used in each data set, indicating where these drew on items from previously validated scales. Internal reliability of measures was generally satisfactory (α > .70). Where reliability fell slightly below this level in either study, reliability of the equivalent measure in the other study was satisfactory. Full details, including sample items, reliability, mean values, and correlations between measures are provided in the online supplemental material. To facilitate comparison of effect sizes, all main measures were standardized prior to analysis.

#### Main outcome measure: Middle childhood externalizing problems

These were measured at age 11 (MCS) or age 9 (FFS) using information from parents, teachers and children. Supplementary analyses in both study samples used additional parent-reported measures gathered at age 3 (as control variables) and in MCS used parent and teacher reports gathered at age 7 (as supplementary outcome measures).

##### MCS

Parent and teacher reports used the combined conduct and hyperactivity/attentional problems five-item subscales from the Strengths and Difficulties Questionnaire (Goodman, 1997). Child reports used items selected for the purposes of this study on delinquent behaviors, school engagement, bullying, and anger.

##### FFS

Parent reports (generally from mothers) used items from the combined aggressive and rule-breaking subscales of the Child Behavior Checklist (Achenbach & Rescorla, 2001). Teacher reports used the externalizing problems subscale of the Social Skills Rating System (Gresham & Elliott, 1990). Child reports used the externalizing subscale of the Self-Description Questionnaire (Marsh, 1990).

#### Primary exposure: Couple supportiveness

Both parents reported on the other’s emotional supportiveness when children were infants (MCS: 9 months; FFS: 12 months). For MCS, parents reported on seven items, including six from the Golombok Rust Inventory of Marital State (Rust, Bennun, Crowe, & Golombok, 1990), concerning the partner’s sensitivity and ability to listen, and the respondent’s feelings of closeness, affection, commitment and happiness in the relationship. For FFS, parents reported on five items concerning the extent to which the partner compromised, listened, empathized, offered encouragement, or expressed affection.

To reflect the dyadic nature of the couple relationship construct, an average of the two parents’ scores was used in the main analysis. Supplementary analyses address the potential relevance of different perceptions of the couple relationship by each parent, which was particularly important given relatively low correlations between ratings of the couple relationship in FFS (*r* = .19, *p* < .001, compared with *r* = .44, *p* < .001 in MCS).

#### Potential mediators

Both parents reported on coparenting and parenting when children were aged 3 (or age 5 for some measures in MCS), described as follows.

##### Coparenting

For MCS, this was based on the average score of one item reported at child aged 3 and 5 by each parent on how often each parent disagreed with their husband/wife/partner over issues relating to the child. For FFS, coparenting was based on six items covering perceived support and discussion of rules, schedules and decisions in raising the child. As for couple supportiveness, the average of both parents’ scores (correlations: MCS, *r* = .44 *p* < .001; FFS, *r* = .25, *p* < .001) was used in the main analyses, supplemented by analyses using individual parent perceptions.

##### Negative parenting

For MCS, this used the eight-item Parent–Child Conflict subscale from the Pianta Child–Parent Relationship Scale (Pianta, 1992). For FFS, this used a measure of harsh discipline, concerning frequency of spanking. (A measure of harsh discipline at ages 3 and 5 (mothers only) was used in supplementary analyses of MCS, for closer comparison with FFS.)

##### Positive parenting

For MCS, this used the seven-item Parent–Child Closeness subscale from the Pianta Child–Parent relationship scale (Pianta, 1992). For FFS, this used the frequency of parental involvement in seven activities with the child, such as reading stories or playing with toys. (Supplementary MCS analyses used a similar age 5 measure of involvement.)

#### Prior measures of mediators

In FFS (but not MCS), coparenting and parenting were also measured at child aged 1.

#### Covariates

These were selected from the literature as potential confounders of associations between couple supportiveness, coparenting, parenting and externalizing problems. Covariates were measured at 9 months (MCS) or at 1 year (FFS). Parental sociodemographic characteristics comprised parents’ ages at the birth of the cohort child, whether parents were married, parents’ ethnicity (MCS: White or ethnic minority; FFS: White non-Hispanic, Black non-Hispanic, Hispanic, and other), and parental education levels. Household characteristics comprised the number of children, whether one or more grandparents lived in the household, whether parents had other children living elsewhere, and income in relation to needs (MCS: household income after tax equivalized for household size and composition; FFS: poverty ratio, i.e., the ratio of total household income before taxation to the U.S. poverty threshold).

Descriptive statistics for sociodemographic covariates are provided in the online supplemental material. For each study, there were statistically significant differences between distributions of covariates in the analysis sample and a complete sample of couples at baseline, although these were small in magnitude (averaging less than a percentage point). For MCS, the analytic sample contained fewer disadvantaged groups (e.g., those with low education or income) than the full couple sample, and fewer ethnic minority groups. For FFS, differences were less consistently associated with disadvantage, with the analytic sample containing more Black and unmarried parents. Compared with FFS, the MCS analysis sample contained fewer unmarried parents and disadvantaged groups (such as parents with no educational qualifications), and was less ethnically diverse. All MCS parents were coresiding for children aged 1 to 5 years. In FFS, 15% of FFS parents were living apart at child age 1, and this increased to 37% by age 5.

### Analytic Strategy

First, we examined levels and patterns of missing information in the analytic samples. For any variable, on average 4% of MCS and 11% of FFS cases contained incomplete information. This was higher for father-reported (averages MCS 4%, FFS, 20%) and teacher-reported information (MCS 27%, FFS 33%) than for mother-and child-reported information (MCS 1%, FFS 2% to 3%). In both samples, incomplete information was more common with younger maternal age, maternal ethnic minority, and lower family income. To reduce bias, missing information was imputed using Mplus Version 7.3 (Muthén & Muthén, 1998–2012). The inclusion in the imputation model of all variables predicting missingness increases the plausibility of the missing at random assumption (White, Royston, & Wood, 2011). To strengthen prediction of missing father-reported measures in FFS, the imputation model included auxiliary variables such as mothers’ reports of fathers’ parenting at baseline. As recommended, the number of imputed data sets (70 for each sample) exceeded the percentage of incomplete cases (White et al., 2011). Our choice of multiple imputation rather than relying on full information maximum likelihood (FIML), another appropriate method available in Mplus to address missing information bias (Graham, 2009), was driven primarily by convenience: for each data set it was simpler to conduct a separate imputation model inclusive of all missingness predictors before running the various main and supplementary analysis models reported here. As expected, checking our final path models using FIML on the original nonimputed analysis samples gave closely similar findings to those using imputed data. With one exception in relation to bootstrapped estimates (see below), which could only be produced using nonimputed data in Mplus, findings using FIML are not reported here.

To address our first hypothesis, we modeled middle childhood externalizing problems as a latent construct indicated by parent-, teacher-, and child-reported information (loadings .5 to .7 in both data sets). Associations between couple supportiveness and middle childhood externalizing problems were assessed, before and after adjusting for sociodemographic covariates. Supplementary analyses assessed the influence of each parent’s views on partner supportiveness, comparing effects found in separate models using mothers’ and fathers’ perceptions using *z* tests of coefficients. In order to put analyses using continuous measures in a clinical perspective, supplementary analyses using MCS explored associations between couple supportiveness divided into tertiles (low, medium, high) and severe externalizing problems scored using recommended cut-offs (Goodman, 1997).

To address our second hypothesis, we used path models to estimate indirect effects of couple supportiveness via coparenting and parenting mediators. Potential mediators were included in stages, corresponding to different elements of the theorized model (see Figure 1): (1) parenting only, (2) coparenting as an intermediary between couple supportiveness and parenting, (3) as (2), with an additional direct path from coparenting to externalizing problems (full theorized model). Model fit was assessed at each stage. Indirect effects in the final model were computed as products of path coefficients. Three Wald tests were used to compare the magnitude of different sets of indirect pathways (coparenting vs. parenting, positive vs. negative parenting, and mothers’ vs. fathers’ parenting). Following recommended practice (MacKinnon, Lockwood, & Williams, 2004), supplementary analyses on nonimputed data produced bias-corrected bootstrapped estimates of indirect effects. (Because of software limitations, the robust maximum likelihood estimator and auxiliary missing information could not be used in bootstrap models.) To provide more robust tests of the direction of effects, we conducted supplementary analyses using some repeated measures to assess mediating pathways after allowing for earlier measurement of externalizing problems (both data sets) or prior values of mediators (FFS only). Supplementary analyses also considered (1) whether indirect effects were similar, depending on whether mothers or fathers provided information on couple supportiveness and coparenting, and (2) whether alternative measures of negative and positive parenting available in MCS produced similar findings. In these analyses, we compared coefficients obtained in different models using *z* tests.

Analyses were performed on imputed data sets using Mplus Version 7.3. For MCS, weights were used to address survey attrition. For FFS, no corresponding weights to address attrition were available. Available cross-sectional weights for FFS were not used since oversampling of unmarried and noncoresiding couples was considered a virtue for assessing consistency of causal mechanisms (though results should not be interpreted as representative of the 20 US cities they were sampled from). Indicator cut-offs applied to assess absolute model fit were <.06 for the root mean square error of approximation (RMSEA) and <.08 for the standardized root mean residual (SRMR; Hu & Bentler, 1999). For path models, comparative fit of models with different sets of indirect pathways was assessed using the Akaike and Bayesian information criteria (AIC and BIC, respectively), with smaller values indicating better fit. To address clustering of observations in the MCS sample design and non-normality of measures in both analytic samples, we used maximum likelihood estimation with robust standard errors. Throughout, statistical significance was defined at the *p* < .05 level. Estimates for regression and path models standardized with respect to predictors and outcomes are described, with unstandardized estimates available in the online supplemental material. Because of software constraints, estimates of indirect effects produced using imputed data are standardized with respect to predictors only.

## Results

In both study samples, couple supportiveness in infancy predicted fewer externalizing problems in middle childhood, with similar effects in both data sets (standardized betas: MCS = age 11, −.15; FFS = age 9, −.14, both *p* < .001, corresponding to the standard deviation reduction in externalizing problems produced by a one standard deviation increase in couple supportiveness). These unadjusted effects were slightly reduced after adjusting for sociodemographic covariates (MCS = age 11, −.13; FFS = age 9, −.11, both *p* < .001, see Table 1). These effects are based on the average of both parents’ reports of partner supportivneness. In supplementary analyses, *z* tests comparing effects based solely on mothers’ or on fathers’ individual reports of partner supportiveness found stronger associations for mothers’ reports (see Table A in the online supplemental material), although this could reflect shared variance (mothers usually provided parent reports of externalizing problems). To assess this, we restricted latent constructs of externalizing problems to teacher and child indicators. In MCS, the difference between the effect of mothers’ and fathers’ reports became nonsignificant, while in FFS mothers’ reports still showed a stronger association. A second supplementary analysis examined the association between different tertiles of couple supportiveness (high, medium, low) and severe levels of externalizing problems at ages 7 and 11, using MCS data. When compared with couples with high supportiveness, low levels of supportiveness increased the odds of externalizing problems that could be clinically significant (see Table B in the online supplemental material).[Table-anchor tbl1]

Turning to our second hypothesis, we used path models to explore coparenting and parenting as potential mediators of the effect of couple supportiveness on externalizing problems (parent, teacher and child reported) at age 11 (MCS) or age 9 (FFS). Sociodemographic covariates were allowed to predict mediators and outcome. We explored model fit by including mediators in stages as previously described. The final stage (corresponding to the conceptual model in Figure 1) had satisfactory absolute fit (MCS: χ^2^[59] = 1345.7 *p* < .001, RMSEA = .06, SRMR = .02; FFS: χ^2^[44] = 157.4 *p* < .001, RMSEA = .04, SRMR = .01) and a better fit than earlier stages (based on lowest AIC and BIC values; see Table C in the online supplemental material). In both studies, the direct effect of couple supportiveness on externalizing problems (i.e., the effect not explained by mediators) was of a similar magnitude (MCS = −.05, *p* < .01; FFS = −.05, *p* = .165). Given that the total effect of couple supportiveness was also similar in both studies, this indicates a similar combined indirect effect of coparenting and parenting (60% of the total effect in MCS, 55% in FFS).

**Figure 2 fig2:**
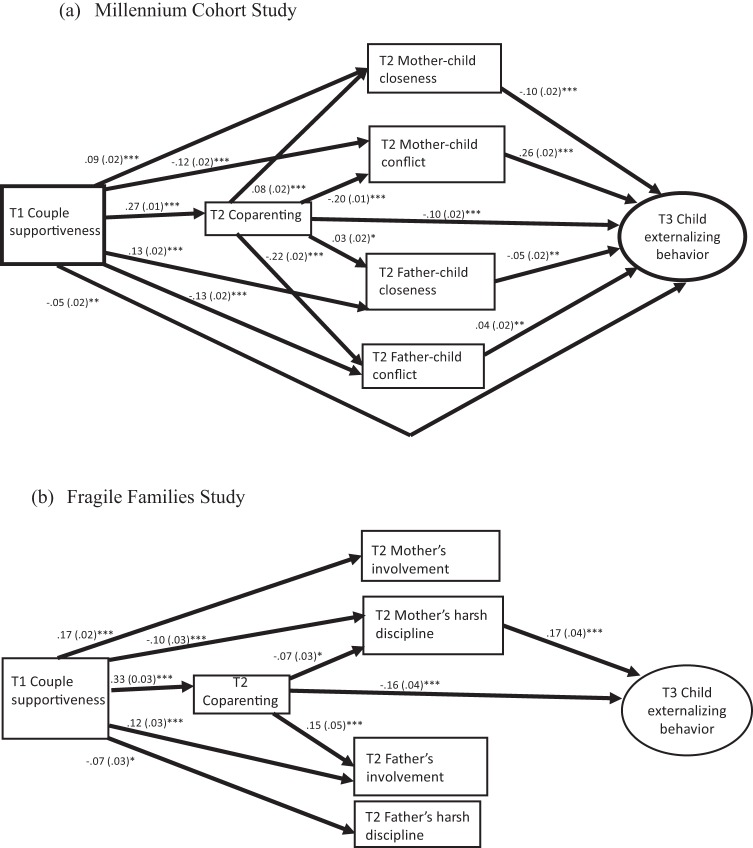
Final path models of associations between couple supportiveness in infancy and middle childhood externalizing problems. (a) Millennium Cohort Study (MCS). (b) Fragile Families Study (FFS). Model fit: (a) MCS: χ^2^(59) = 1345.7 *p* < .001, root mean square error of approximation (RMSEA) = .06, standardized root mean residual (SRMR) = .02; (b) FFS: χ^2^(44) = 157.4 *p* < .001, RMSEA = .04, SRMR = .01. Child ages at T1 = 9 months (MCS), 1 year (FFS), T2 = 3/5 years (MCS), 3 years (FFS), T3 = 11 years (MCS), 9 years (FFS). Models adjusted for parents’ age, ethnicity, education, marital status, number of children, nonresident children, resident grandparent, and household income. Correlations modeled between T2 parenting measures, and all nonsignificant associations between constructs, have been omitted. Figures show standardized coefficients with standard errors in parentheses. * *p* < .05. ** *p* < .01. *** *p* < .001.

Figure 2 shows statistically significant paths between exposure, mediators and outcome in the final path model for each study sample. Indirect effects are listed in full in Table 2 (with bias-corrected bootstrap confidence intervals produced using nonimputed data in the online supplemental material; see Tables D and E). In MCS, there were significant pathways via all mediators, although in FFS the only significant paths were via coparenting and/or mother’s negative parenting. Table 2 also shows the results of Wald tests comparing different sets of pathways. In FFS, paths involving coparenting were stronger than those involving parenting alone, although in MCS there was no difference. In both data sets, pathways via negative parenting were stronger than via positive parenting, and paths were stronger for mothers’ than for fathers’ parenting.[Fig-anchor fig2][Table-anchor tbl2]

In both study samples, further models allowed for parent-reported externalizing problems at child age 3. This provided a test of whether coparenting and parenting mediated effects of couple supportiveness on changes in externalizing problems from preschool age to age 11 (MCS) or age 9 (FFS). After allowing for concurrent externalizing problems, all paths via coparenting and/or mother’s negative parenting remained significant in both data sets (see Table F in the online supplemental material). In FFS, a further model allowing for coparenting and parenting at child age 1 provided a more robust test of whether couple supportiveness at age 1 produced a change in mediators at age 3. In this model, paths via coparenting alone and mother’s negative parenting alone both remained statistically significant (see Figure A and Table G in the online supplemental material). Two sets of supplementary analyses assessed the sensitivity of indirect effects to the source of information, and to the different parenting measures used in the two studies. Results showed a high degree of consistency. In relation to source of information, there were significant indirect pathways involving coparenting (with or without parenting), regardless of whether fathers or mothers supplied information on couple supportiveness and coparenting (see Table H in the online supplemental material). In the MCS data set (but not the FFS data set), *z* tests showed combined indirect pathways were larger in magnitude when mothers’ perceptions of supportiveness and coparenting were used. Supplementary MCS analyses substituted measures of parental involvement (age 5) for parent–child closeness (age 3), and mother’s use of harsh discipline (ages 3 and 5) for mother–child conflict. As in the main model (see Table 2), significant indirect pathways were found via positive and negative parenting, indicating broad equivalence of alternative study parenting measures (see Table I in the online supplemental material). Nonetheless, *z* tests showed that paths via alternative MCS parenting measures were reduced in size. This suggests that in the MCS data set, involvement and harsh discipline measures (equivalent to the FFS parenting measures) did not provide such a sensitive test of the effect of couple supportiveness on parenting, as compared with measures of parent–child closeness and conflict.

## Discussion

This study found that more supportive couple relationships in early childhood reduced externalizing problems in middle childhood (including reduced risk of clinically significant problems). Much of the effect of couple supportiveness was explicable in terms of coparenting and parenting behavior during preschool years. This is the first longitudinal study finding spillover pathways to middle childhood adjustment from couple supportiveness in infancy via both coparenting and parenting. It extends our understanding of spillover involving coparenting, from previous cross-sectional work (O’Leary & Vidair, 2005; Stroud et al., 2015). It confirms the relevance of parenting spillover to effects of positive relationship qualities, previously documented for couple conflict (Gerard et al., 2006; Lindsey et al., 2009; Rhoades et al., 2011; Schoppe-Sullivan et al., 2007; Stover et al., 2016). Strengths of the study include the use of information from both parents to assess relationship quality, parenting and coparenting; outcome information from parents, teachers and children to reduce effects of shared method variance; and allowance for confounding. A broadly consistent pattern of results across two population-based samples from different contexts, with the U.S. sample incorporating more unmarried and separated parents than previously studied, permits greater generalizability of study findings.

The total effect size of couple supportiveness was similar in magnitude across the two study samples, although smaller than effects typically found elsewhere (Buehler et al., 1997; Reid & Crisafulli, 1990). This could be because our meaure of couple supportiveness focused on more positive aspects of the couple relationship rather than on marital conflict or physical violence: our effect sizes are closer to those of a meta-analysis that included measures of general marital distress as well as conflict (.16; Reid & Crisafulli, 1990) than to effects from a meta-analysis focusing specifically on conflict (.32; Buehler et al., 1997). Previous studies have also relied heavily on cross-sectional data, compared with our 8- to 10-year follow-up period.

Our findings in relation to mediators strengthen the limited existing evidence for spillover effects of the couple relationship onto two types of family process linked with externalizing problems during middle childhood (O’Leary & Vidair, 2005; Stroud et al., 2015). Pathways involving coparenting were more important (U.S.) or just as important (U.K.) as those mediated by parenting alone. The greater importance of coparenting in the U.S. study may reflect superior measurement, set against better measures of parenting in the U.K. study. Overall, our study confirms previous cross-sectional work viewing coparenting as an intermediary between the couple relationship and parenting (O’Leary & Vidair, 2005). It also supports cross-sectional work indicating that coparenting transmits additional effects of the couple relationship on children’s behavior, over and above those conveyed via parenting (Stroud et al., 2015). For young children, triadic coparenting disagreements may be more salient than similar, more direct, effects of dyadic couple interactions: The child is likely to be present and sensitive to matters concerning him/herself (Feinberg, 2003; Talbot & McHale, 2004).

In both study samples, spillover pathways found via negative parenting echo findings from other studies of families with young children, via parental harsh or inconsistent discipline, hostility, overreactivity, and psychological control (Gerard et al., 2006; O’Leary & Vidair, 2005; Rhoades et al., 2011; Schoppe-Sullivan et al., 2007; Stover et al., 2016). In the U.K. sample, there were also smaller pathways involving positive parenting. This, too, supports other work on families with young children finding pathways via positive parenting, including warmth, involvement, emotional reciprocity and attachment security (Gerard et al., 2006; Lindsey et al., 2009; Schoppe-Sullivan et al., 2007). The stronger effect of negative parenting compared with positive parenting we found overall might reflect our available measures, but is striking given that our primary exposure of couple supportiveness did not specifically measure couple conflict. It chimes with a population study of marital conflict, which also found larger paths to externalizing problems via negative parenting (Gerard et al., 2006).

Stronger pathways were found for mothers’ than for fathers’ parenting in both samples. This might reflect higher levels of missing information for fathers, and/or shared method variance inflating associations between maternal parenting and mother-reported child externalizing problems. Nonetheless, we took precautions to guard against both these risks, in strengthening imputation of missing father information with auxiliary information and using multiinformant outcome measures. Our findings contrast with stronger effects for fathers’ than mothers’ hostile parenting found in two studies (Rhoades et al., 2011; Stover et al., 2016) and another study finding no difference (Stroud et al., 2015); but these studies used couple-based convenience samples, which may overrepresent families where fathers are more involved in parenting. Our findings from population-based samples may reflect a more typical picture of the mother as the child’s main caregiver.

Although this study lends strong empirical support to spillover mechanisms, including both parenting and coparenting, the mediators explored here did not fully explain associations between couple relationship quality and child externalizing problems. Moreover, these observational associations only indicate causal effects under the assumption of no unmeasured confounding. Despite the use of several strategies to strengthen causal inference, unmeasured confounding remains a possibility, for example, from parental temperament. Last, although analyses using repeated measures supported the idea of a directional effect from the couple relationship to child adjustment via mediators, our study design does not allow for the likely bidirectionality of associations between measures over time indicated by other studies (e.g., Goldberg & Carlson, 2014). Further work is needed to address these issues, using repeated measurements to construct fixed effects and cross-lagged models, or using marginal structural models to adjust for time-varying confounding.

Limitations in addition to those already noted include reliance on parents for sensitive information subject to social desirability and other biases and use of mediator measures that may not adequately capture underlying constructs. Notably, coparenting measures did not fully encompass the multidimensional nature of coparenting theorized elsewhere (Feinberg, 2003) and were not previously validated in either data set. The study samples differed in measures used for various constructs and the age of children at the final outcome, making it is difficult to determine what causes between-study differences (differences in measurement, sampling, context, etc.). Measurement limitations of this type are common when comparing across large multipurpose population samples, though this is counterbalanced by already-noted strengths including generalizability and consistency across different contexts. However, the FFS sample is not fully representative of the United States, so analyses should not be interpreted as a straight U.S.–U.K. comparison.

Overall, our study supports a broad model of spillover effects on child adjustment, operating consistently across different settings. Future research using population-based samples would benefit from more detailed measures of the couple relationship, including both positive and negative dimensions, and a fuller assessment of family processes. It is reasonable to assume that our coparenting, and parenting measures gathered at preschool and school transition reflected earlier as well as concurrent processes (Dallaire & Weinraub, 2005; Laxman et al., 2013), but future work should use earlier measurements to better assess impacts on infant and toddler development. Our own study suggests a particular need to explore direct effects of coparenting in more detail. Our measures did not capture whether the child was present during coparenting, but future work could examine young children’s psychological responses to covert versus overt coparenting tensions, explore possible moderators and investigate additional aspects of children’s adjustment.

Our study offers greater clarity on the contribution of coparenting to causal mechanisms linking the overall couple relationship in the important vulnerable period of early childhood to later child behavior. The findings lend strong support to parenting interventions that include coparenting as well as dyadic couple relationship and individual parenting skills. In recent years there has been a surge of interest in interventions specifically designed to help couples in the early stages of parenthood manage the stresses and strains involved in child rearing, by developing their coparenting skills (Pruett, Pruett, Cowan, & Cowan, 2017). The transition to parenthood may be an ideal opportunity to intervene to help families, being a period when couples are receptive to advice and are less likely to have well-established patterns of coparenting (Feinberg, 2002). Interventions to facilitate coparenting, though presenting many challenges for couples with unstable relationships (McHale, Waller, & Pearson, 2012), also may be more attractive to parents than relationship counseling (Doss, Cicila, Hsueh, Morrison, & Carhart, 2014).

## Supplementary Material

10.1037/fam0000492.supp

## Figures and Tables

**Table 1 tbl1:** Regression Models of Associations Between Couple Supportiveness and Middle Childhood Externalizing Problems (Standardized Estimates)

		Unadjusted			Adjusted		
Measure (reference group)	Contrast	β	*SE*	*p*	β	*SE*	*p*
Millennium Cohort Study (*N* = 5,779)							
		Age 11 externalizing problems					
Couple supportiveness		–.15	.02	<.001	–.13	.02	<.001
Mother’s age					–.07	.02	.005
Father’s age					–.06	.02	.014
Number of children					.05	.02	.009
Household income					–.01	.02	.598
Married at birth					–.06	.02	.002
Grandparent in household					–.01	.02	.579
Mother has children elsewhere					.02	.02	.238
Father has children elsewhere					.06	.02	.003
Mother’s education (NVQ 4–5)	NVQ Level 2–3				.06	.02	.003
	NVQ Level 1				.05	.02	.014
	None				.11	.03	<.001
Father’s education (NVQ 4–5)	NVQ Level 2–3				–.07	.02	.002
	NVQ Level 1				–.08	.02	<.001
	None				.03	.02	.087
Mother’s ethnic group (White)	Indian				–.02	.02	.145
	Pakistani/Bangladeshi				–.02	.02	.299
	Black				.00	.02	.860
	Other				–.04	.02	.015
Father’s ethnicity different					.01	.02	.566
Fragile Families Study (*N* = 2,069)							
		Age 9 externalizing problems					
Couple supportiveness		–.14	.03	<.001	–.11	.03	<.001
Mother’s age					–.12	.05	.015
Father’s age					–.01	.05	.890
Number of children					–.02	.03	.470
Income to poverty ratio					–.07	.03	.044
Married at birth					–.04	.04	.279
Grandparent in household					.04	.03	.261
Mother has children elsewhere					.01	.03	.799
Father has children elsewhere					.09	.04	.011
Mother’s education (none)	High school				–.06	.04	.121
	Some college				–.12	.04	.002
	College				–.12	.04	.007
Mother’s ethnic group (White)	Black				.04	.05	.369
	Hispanic				–.17	.04	<.001
	Other				–.02	.03	.421
Father’s ethnicity different					–.05	.03	.095
Father more education than mother					–.04	.03	.176
*Note.* NVQ refers to National Vocational Qualifications framework: Levels 4 and 5 correspond to university-level qualifications (for more details see https://www.gov.uk/what-different-qualification-levels-mean/list-of-qualification-levels). *SE* = standard error.							

**Table 2 tbl2:** Indirect Effects From Couple Supportiveness to Children’s Externalizing Problems

Mediator(s)	Effect	*SE*	*p*	Mediator(s)	Effect	*SE*	*p*
	Millennium Cohort Study (child age 11)				Fragile Families Study (child age 9)		
Coparenting only	–.029	.005	<.001	Coparenting only	–.054	.015	<.001
Coparenting and mother–child closeness	–.002	.001	<.001	Coparenting and mother’s involvement	.000	.001	.978
Coparenting and mother–child conflict	–.015	.002	<.001	Coparenting and mother’s harsh discipline	–.004	.002	.028
Coparenting and father–child closeness	.000	.000	.108	Coparenting and father’s involvement	.001	.002	.475
Coparenting and father–child conflict	–.003	.001	.010	Coparenting and father’s harsh discipline	.001	.001	.493
Mother–child closeness only	–.010	.002	<.001	Mother’s involvement only	.000	.006	.980
Mother–child conflict only	–.035	.005	<.001	Mother’s harsh discipline only	–.016	.006	.005
Father–child closeness only	–.007	.003	.007	Father’s involvement only	.004	.005	.454
Father–child conflict only	–.006	.002	.012	Father’s harsh discipline only	–.003	.003	.443
All via mother’s parenting	–.063	.006	<.001	All via mother’s parenting	–.020	.008	.015
All via father’s parenting	–.016	.004	<.001	All via father’s parenting	.003	.007	.654
All via coparenting (+/−parenting)	–.052	.006	<.001	All via coparenting (+/−parenting)	–.055	.015	<.001
All via parenting only	–.058	.007	<.001	All via parenting only	–.016	.009	.089
All via positive parenting	–.019	.004	<.001	All via positive parenting	.005	.009	.574
All via negative parenting	–.060	.006	<.001	All via negative parenting	–.022	.006	<.001
Comparison of pathways	Wald test	*df*	*p*	Comparison of pathways	Wald test	*df*	*p*
Mother’s vs. father’s parenting	68.73	1	<.001	Mother’s vs. father’s parenting	4.00	1	.045
Coparenting (+/−parenting) vs. parenting only	.92	1	.338	Coparenting (+/−parenting) vs. parenting only	4.80	1	.029
Positive vs. negative parenting	32.29	1	<.001	Positive vs. negative parenting	5.75	1	.017
*Note.* These represent indirect effects in the final path models. Effects are standardized with respect to predictors only. *SE* = standard error.
