# Supplementary material for: Coparenting and Parenting Pathways From the Couple Relationship to Children’s Behavior Problems
Source: J Fam Psychol. 2018 Dec 27;33(2):215–25. doi: 10.1037/fam0000492 (PMC6388648; doi:10.1037/fam0000492)
Supplement: Supplementary file 1 [file FAM-2018-1474Suppl.zip › JFP rev1 Online Supplemental File S1 revised.pdf]

Online Supplemental File S1 Detailed information on Main measures

Table 1 Details of main measures used in the Millennium Cohort Study (MCS) and Fragile Families Study (FFS)

Construct	Study	Child age	Source(s) of information	Number of Items, reliability	Sample items	Response scale(s)
Externalising problems	MCS	3, 7 11	Parent	10, alphas=0.78-0.81	For conduct problems: "fights or bullies other children", "Often has temper tantrums"; for hyperactivity/attentional problems: "Easily distracted", "Sees tasks through to the end"(reversed).	3-point: 0 'not true', 1 'somewhat true', 2 'certainly true'
		7, 11	Teacher	10, alphas=0.87	As above	As above
		11	Child	6, standardised alpha=0.67	Delinquent behaviours ever engaged in from a list of five items (shoplifting, being noisy or rude in public, making graffiti in a public place, wilful damage to property, truancy); school engagement using 2 items (how often do you "misbehave or cause trouble in class?", "try your best at school"(reversed)), bullying (2 items concerning how often do you "hurt or pick on" siblings, other children) and anger (how often were you angry in the last four weeks?).	Delinquent behaviours were binary items (total score range 0-5). School engagement items used a 4-point scale (all of the time, most of the time, some of the time, never), bullying used a 6-point scale (most days, about once a week, about once a month, every few months, less often, never) and anger a 5-point scale (never, almost never, sometimes, often, almost always)
	FFS	3, 9	Parent	22, 35 alphas=0.84-91	For aggressive behavior: "argues a lot", "is cruel, bullies or shows meanness to others"; for rule-breaking: "drinks alcohol without parents' approval", "doesn't seem to feel guilty after misbehaving".	3-point scale (not true, somewhat or sometimes true, very true or often true)

Construct	Study	Child age	Source(s) of information	Number of Items, reliability	Sample items	Response scale(s)
		9	Teacher	6, alpha=0.92	“Fights with others”, “threatens or bullies others”.	4-point scale (never, sometimes, often, very often)
		9	Child	6, alpha=0.76	“I get distracted easily”, “I get into trouble for fighting with other kids”	4-point scale: not at all true, a little bit true, mostly true, very true.
Couple supportiveness	MCS	9 months	Mother, Father	7, standardised alphas: mothers=0.82, fathers =0.79	From the Golombok Rust Inventory of Marital State: “usually sensitive to and aware of my needs”, “doesn't seem to listen to me". Additional item : "Please enter the number which best describes how happy or unhappy you are with your relationship, all things considered"	5-point scale from (strongly agree” to “strongly disagree). Scoring for additional item on a 7 point linear scale from 1 “very unhappy” to 7 “very happy”
	FFS	1 year	Mother, Father	5 items (alphas: mothers=0.74, fathers=0.67	“fair and willing to compromise when you have a disagreement”; “listens to you when you need someone to talk to”	3-point scale (often, sometimes, never).
Coparenting	MCS	3,5	Mother, Father	1, alphas (repeated scores): father=0.62, mother=0.61	"how often do you disagree with (husband/wife/ partner) over issues relating to (child)?"	6-point scale: (more than once a day, once a day, several times a week, once a week, less than once a week, never).
	FFS	1, 3	Mother, Father	6, alphas mothers 0.70-85, fathers 0.60-72	“respects the schedules and rules you make for (child)”, “supports you in the way you want to raise (child)”, “you can trust (partner) to take good care of (child)”.	Age 1: 3-point scale: (always true, sometimes true, rarely true); age 3: 4-point scale ((always true, sometimes true, rarely true, never true);
Positive parenting	MCS	3 (parent-child closeness)	Mother, Father	7, alphas mother 0.70, father 0.68	“(child) spontaneously shares information”, “when I praise (child), he/she beams with pride”	5-point scale (definitely does not apply, not really, neutral, applies sometimes, definitely applies)

Construct	Study	Child age	Source(s) of information	Number of Items, reliability	Sample items	Response scale(s)
		5 (parental involvement)	Mother, Father	7, alphas mothers=0.72, fathers =0.73	"How often do you…(sample items) read stories to (child), tell stories to (child) not from a book, play with toys or games indoors with (child) ?	6-point scale (every day, several times a week, once or twice a week, once or twice a month, less often, not at all)
	FFS	1, 3 (parental involvement)	Mother, Father	6,7 alphas mother 0.63,72, father 0.73, 0.78	How many days a week do you usually…(sample items): read stories to (child), tell stories to (child), hug or show physical affection to (child), play inside with toys such as blocks or Legos with child ?	7-point scale (0-7 days per week)
Negative parenting	MCS	3 (parent-child conflict)	Mother, Father	8, alphas: mother=0.86, father=0.74	"(Child) and I always seem to be struggling with each other", "(child) remains angry after discipline"	5-point scale (definitely does not apply, not really, neutral, applies sometimes, definitely applies)
		3,5 (harsh discipline)	Mother	1 alpha, repeated mother=0.66	How often do you do the following when (child) is naughty?: smack him/her	5-point scale (never, rarely, once a month, once a week or more, daily)
	FFS	1, 3 (harsh discipline)	Mother, Father (self-reported, and at age 3, report of partner's behaviour)	2, alphas (age 3): mother=0.74, father=0.80	"In the past month, have (you, partner) spanked (Child) because (he/she) was misbehaving or acting up?", (if yes) "Did (you, partner) do this . . every day or nearly every day, a few times a week, a few times this past month, only once or twice?"	5-point scale (every day or nearly every day, a few times a week, a few times this past month, only once or twice, not at all)

Table 2 Descriptive statistics and Pairwise correlations among partner relationship quality, child externalising problems and parenting in the two analytic samples

(a) UK Millennium Cohort Study

		Min	Max	Mean (SE)	1	2	3	4	5	6	7	8	9	10
1	couple supportiveness 9 months	-2.84	1.07	0.04 (0.01)	1.00									
2	externalising age 3 (parent)	0	20	6.29 (0.06)	-0.17	1.00								
3	externalising age 7 (parent)	0	20	4.25 (0.06)	-0.15	0.52	1.00							
4	externalising age 11 (parent)	0	20	4.05 (0.06)	-0.17	0.46	0.66	1.00						
5	externalising age 11 (teacher)	0	20	2.25 (0.06)	-0.06	0.22	0.35	0.42	1.00					
6	externalising age 11 (child)	-0.84	4.51	0.01 (0.01)	-0.07	0.16	0.20	0.27	0.29	1.00				
7	Co-parenting age 3/5	1	6	5.10 (0.01)	0.27	-0.19	-0.16	-0.18	-0.06	-0.07	1.00			
8	Mother-child closeness age 3	1	5	4.80 (0.01)	0.13	-0.12	-0.10	-0.11	-0.03	-0.06	0.04	1.00		
9	Mother-child conflict age 3	1	5	2.27 (0.01)	-0.17	0.56	0.34	0.31	0.06	0.12	-0.22	-0.08	1.00	
10	Father-child closeness age 3	1	5	4.64 (0.01)	0.15	-0.07	-0.07	-0.06	-0.01	0.00	0.05	0.30	-0.05	1.00
11	father –child conflict age 3	1	5	2.39 (0.75)	-0.20	0.28	0.20	0.17	0.05	0.10	-0.26	-0.01	0.32	-0.08

(b) US Fragile Families and Child Well-being Study

		Min	Max	Mean (SD)	1	2	3	4	5	6	7	8	9
1	couple supportiveness age 1	1	3	2.67 (0.27)	1								
2	externalising age 3 (parent)	0	20	5.82 (3.27)	-0.12	1							
3	externalising age 9 (parent)	0	20	1.61 (1.80)	-0.10	0.37	1						
4	externalising age 9 (teacher)	6	24	8.80 (3.72)	-0.07	0.2	0.37	1					
5	externalising age 9 (child)	0	18	5.15 (4.16)	-0.07	0.24	0.34	0.36	1				
6	Co-parenting age 3	1	3	2.76 (0.29)	0.36	-0.15	-0.19	-0.11	-0.13	1			
7	mother involvement age 3	0	8	5.28 (1.36)	0.18	-0.12	-0.04	-0.07	-0.02	0.1	1		
8	mother harsh discipline age 3	1	5	1.95 (1.07)	-0.13	0.2	0.17	0.08	0.15	-0.15	-0.14	1	
9	father involvement age 3	0	8	4.59 (1.53)	0.17	-0.01	-0.02	0.00	0.00	0.14	0.2	-0.05	1
10	father harsh discipline age 3	1	5	1.80 (1.00)	-0.10	0.11	0.08	0.08	0.05	-0.04	-0.06	0.33	-0.08

Note: SE =linearized standard error, SD=Standard deviation.
