# Supplementary material for: Coparenting and Parenting Pathways From the Couple Relationship to Children’s Behavior Problems
Source: J Fam Psychol. 2018 Dec 27;33(2):215–25. doi: 10.1037/fam0000492 (PMC6388648; doi:10.1037/fam0000492)
Supplement: Supplementary file 1 [file FAM-2018-1474Suppl.zip › JFP rev2 Online supplemental File S2 .pdf]

Supplementary File S2 Descriptive statistics showing comparison of study analytic samples with cohort samples of all couples with infants

	Millennium Cohort Study (MCS), UK			Fragile Families and Child Wellbeing Study (FFS), US		
		**Analytic sample (*N* = 5779)**	**Couple families with 9 month old child (*N* = 12,957)[1]**		**Analytic sample (*N* = 2069)**	**Couple families with 1 year old child (*N* = 2928)**
		% or mean (SE)	% or mean (SE)		% or mean (SE)	% or mean (SE)
Child gender[b, ns]	Female	49.9	48.5	Female	48.1	47.8
	Male	50.1	51.5	Male	51.9	52.3
Mother <20 years at birth[b, ns]	yes	2.0	4.2	yes	15.4	15.0
Father <20 years at birth[b, ns]	yes	<1.0	1.2	yes	7.1	7.0
Mother's education[b, ns]	Degree	45.0	44.4	College	14.1	14.8
	Below degree	49.2	48.1	Below college	56.6	55.3
	No qualifications	5.7	7.4	No qualifications	29.3	29.9
Father's education[a,a]	Degree	40.1	39.0	College	12.7	13.9
	Below degree	50.3	50.6	Below college	57.6	56.5
	No qualifications	9.6	10.4	No qualifications	29.7	29.6
Mother's ethnic group[ns, c]	White	91.2	90.5	White, non-Hispanic	25.4	25.8

		Millennium Cohort Study (MCS), UK		Fragile Families and Child Wellbeing Study (FFS), US		
		**Analytic sample (*N* = 5779)**	**Couple families with 9 month old child (*N* = 12,957)[1]**		**Analytic sample (*N* = 2069)**	**Couple families with 1 year old child (*N* = 2928)**
		% or mean (SE)	% or mean (SE)		% or mean (SE)	% or mean (SE)
	Black/Black British	1.3	1.5	Black, non-Hispanic	43.4	40.8
	Pakistani/Bangladeshi	3.3	3.6	Hispanic	27.6	29.0
	Indian	2.3	2.0	Other	3.6	4.3
	Other	1.9	2.4			
Father's ethnic group[a, c]	White	90.9	90.0	White, non-Hispanic	22.9	23.6
	Black/Black British	1.4	2.0	Black, non-Hispanic	45.8	42.8
	Pakistani/Bangladeshi	3.2	3.7	Hispanic	27.4	29.1
	Indian	2.4	2.0	Other	4.0	4.5
	Other	2.1	2.3			
Marital status [c,b]	Unmarried	23.4	27.9	Unmarried	66.2	64.5
	Married	76.9	72.1	Married	33.8	35.3
Household income[b, ns]	<60% median (OECD indicator)	13.0	18.8	At or below US poverty threshold	31.8	31.4
Parents cohabiting[ns,ns]	Child aged 1	100	100	Child aged 1	84.6	85.4

		Millennium Cohort Study (MCS), UK			Fragile Families and Child Wellbeing Study (FFS), US	
		**Analytic sample (*N* = 5779)**	**Couple families with 9 month old child (*N* = 12,957)[1]**		**Analytic sample (*N* = 2069)**	**Couple families with 1 year old child (*N* = 2928)**
		% or mean (SE)	% or mean (SE)		% or mean (SE)	% or mean (SE)
Children in household[a, ns]	one	41.7	42.0	one	30.7	30.8
	two	39.1	37.6	two	35.8	35.2
	three or more	19.3	20.4	three or more	33.6	34.0
Mother has children living elsewhere[ns,a]	yes	3.2	3.3	yes	6.0	6.3
Father has children living elsewhere[b,ns]	yes	11.8	12.8	yes	26.9	25.9
Grandparent resident in household[a,ns]	yes	3.1	3.4		15.4	15.7

Note: figures for MCS samples use baseline survey weights; figures for FFS are unweighted. [1]Sample includes all couple families in all United Kingdom countries included in the survey (England, Scotland and Wales). Superscripts in column 1 indicate statistical differences in distribution between the analytic sample and full couple sample for each study, with the MCS shown first and FFS second: superscript a denotes p <0.05, superscript b p <0.01, superscript c p <0.001, and superscript ns = not significant.
