# Supplementary material for: Coparenting and Parenting Pathways From the Couple Relationship to Children’s Behavior Problems
Source: J Fam Psychol. 2018 Dec 27;33(2):215–25. doi: 10.1037/fam0000492 (PMC6388648; doi:10.1037/fam0000492)
Supplement: Supplementary file 1 [file FAM-2018-1474Suppl.zip › JFP rev2 Online supplemental file S3 .pdf]

Online supplemental file S3 Supplementary analyses

Table A Associations between couple supportiveness and middle childhood externalizing problems, according to source of information on supportiveness and externalizing problems

	**Outcome: Externalizing problems based on 3 indicators (parent, teacher, child)**							
	Model A effect: Mother-reported supportiveness			Model B effect: Father-reported supportiveness			Model A vs B comparison	
Study sample	beta	SE	*p*	beta	SE	*p*	z	*p*
Millennium Cohort Study	-0.15	0.02	<.001	-0.07	0.02	<.001	-3.47	<.001
Fragile Families Study	-0.14	0.03	<.001	-0.02	0.03	0.514	-2.76	0.006
	**Outcome: Externalizing problems based on 2 indicators (teacher, child)**							
Study sample	Model C effect: Mother-reported supportiveness			Model D effect: Father-reported supportiveness			Model C vs D comparison	
	beta	SE	*p*	beta	SE	*p*	z	*p*
Millennium Cohort Study	-0.05	0.02	0.037	-0.01	0.02	0.518	-1.26	0.208
Fragile Families Study	-0.12	0.03	<.001	-0.01	0.04	0.733	-2.17	0.030

Note: Models controlled for parents' ages, ethnicity, education, marital status, number of children, children living elsewhere, resident grandparent and household income (MCS equivalised, FFS income to poverty ratio). Effects are standardized with respect to predictors and outcomes. SE = standard error.

Table B Associations between level of couple supportiveness and severe externalizing problems (Millennium Cohort Study)

		**Age 7 borderline abnormal/abnormal problems**											
		Parent-reported (*N* = 5220)						Teacher-reported (*N* = 3522)					
		Unadjusted			Adjusted			Unadjusted			Adjusted		
		OR	95% CI	*p*	OR	95% CI	*p*	OR	95% CI	*p*	OR	95% CI	*p*
Couple supportiveness tertiles (good)	medium	1.71	1.31, 2.22	<.001	1.61	1.22, 2.14	0.001	1.14	0.89, 1.46	0.304	1.07	0.83, 1.39	0.611
	poor	2.22	1.72, 2.86	<.001	1.99	1.52, 2.59	<.001	1.49	1.19, 1.88	<.001	1.32	1.04, 1.67	0.025
		**Age 11 borderline abnormal/abnormal problems**											
		Parent-reported (*N* = 5367)						Teacher-reported (*N* = 4023)					
		Unadjusted			Adjusted			Unadjusted			Adjusted		
		OR	95% CI	*p*	OR	95% CI	*p*	OR	95% CI	*p*	OR	95% CI	*p*
Couple supportiveness tertiles (good)	medium	1.58	1.21, 2.04	0.001	1.47	1.12, 1.92	0.005	1.16	0.85, 1.57	0.347	1.08	0.79, 1.47	0.638
	poor	1.95	1.48, 2.56	<.001	1.67	1.25, 2.22	0.001	1.23	0.91, 1.64	0.174	1.15	0.86, 1.54	0.332

Note: OR = odds ratio, CI = confidence interval. Adjusted models controlled for parents' ages, ethnicity, education, marital status, number of children, children living elsewhere, resident grandparent and household income.

Table C Model fit of different path models

Data set	Model	AIC	BIC
Millennium Cohort Study	Stage 1	145995.6	146988.2
	Stage 2	145620.6	146639.9
	Stage 3	145582.4	146608.3
Fragile Families Study	Stage 1	59118.87	59857.03
	Stage 2	59054.52	59815.22
	Stage 3	59032.03	59798.37

AIC = Akaike information criterion, BIC = Bayesian information criterion**.** Stage 1: parenting mediators only, Stage 2: coparenting added as intermediate mediator between couple supportiveness and parenting, Stage 3 (final): path from coparenting directly to externalizing problems added to stage 2 model.

Table D Indirect effects from couple supportiveness to children's externalizing problems at 11 years in the UK Millennium Cohort Study: bias-corrected bootstrap estimates and confidence intervals

	Standardized with respect to predictors			Standardized with respect to predictors and outcome		
**Mediator(s)**	Estimate	95% CI		Estimate	95% CI	
Coparenting only	**-0.040**	**-0.028**	**-0.016**	**-0.036**	**-0.025**	**-0.015**
Coparenting and mother-child closeness	**-0.005**	**-0.003**	**-0.002**	**-0.004**	**-0.003**	**-0.002**
Coparenting and mother-child conflict	**-0.021**	**-0.016**	**-0.013**	**-0.018**	**-0.014**	**-0.011**
Coparenting and father-child closeness	-0.002	-0.001	0.000	-0.002	-0.001	0.000
Coparenting and father-child conflict	**-0.006**	**-0.003**	**-0.001**	**-0.005**	**-0.003**	**-0.001**
Mother-child closeness	**-0.014**	**-0.008**	**-0.002**	**-0.013**	**-0.007**	**-0.002**
Mother-child conflict	**-0.047**	**-0.035**	**-0.025**	**-0.042**	**-0.031**	**-0.022**
Father-child closeness	**-0.018**	**-0.011**	**-0.006**	**-0.016**	**-0.009**	**-0.005**
Father-child conflict	**-0.012**	**-0.006**	**-0.002**	**-0.011**	**-0.005**	**-0.001**

Note: Figures in bold type show statistically significant indirect effects. CI = confidence interval. Model controlled for parents' ages, ethnicity, education, marital status, number of children, children living elsewhere, resident grandparent and household income. Estimates were produced using the MCS analysis sample, with missing data handled using Full Information Maximum Likelihood and a maximum likelihood estimator, using complex survey features including longitudinal survey weights to conduct bootstrapping, see Asparouhov, T. and B. Muthén (2010) Resampling Methods in Mplus for Complex Survey Data https://www.statmodel.com/download/Resampling_Methods5.pdf. Estimates shown are not directly comparable with estimates shown in Table 2, which were produced using multiply imputed data sets and a robust maximum likelihood estimator, allowing for complex survey features.

Table E Indirect effects from couple supportiveness to children's externalizing problems at 9 years in the US Fragile Families Study: bias-corrected bootstrap estimates and confidence intervals

	Standardized with respect to predictors			Standardized with respect to predictors and outcome		
**Mediator(s)**	Estimate	95% CI		Estimate	95% CI	
Coparenting only	**-0.067**	**-0.029**	**-0.114**	**-0.061**	**-0.028**	**-0.102**
Coparenting and mother's involvement	0.000	0.002	-0.001	0.000	0.002	-0.001
Coparenting and mother's harsh discipline	**-0.002**	**-0.012**	**-0.006**	**-0.006**	**-0.029**	**-0.015**
Coparenting and father's involvement	0.001	0.006	-0.003	0.001	0.006	-0.003
Coparenting and father's harsh discipline	0.004	-0.001	0.000	0.002	-0.014	-0.003
Mother's involvement	0.001	0.011	-0.008	0.001	0.009	-0.007
Mother's harsh discipline	**-0.017**	**-0.007**	**-0.032**	**-0.015**	**-0.006**	**-0.029**
Father's involvement	0.002	0.013	-0.008	0.002	0.012	-0.007
Father's harsh discipline	-0.004	0.002	-0.016	-0.003	0.002	-0.014

Note: Figures in bold type show statistically significant indirect effects. CI = confidence interval. Model controlled for parents' ages, ethnicity, education, marital status, number of children, children living elsewhere, resident grandparent and household income to poverty ratio. Estimates were produced using the FFS analysis sample, with missing data handled using Full Information Maximum Likelihood and a maximum likelihood estimator. Estimates are not directly comparable with estimates shown in Table 2, which were produced using multiply imputed data sets and a robust maximum likelihood estimator.

Table F Indirect effects from couple supportiveness to middle childhood externalising problems, allowing for pre-school age problems

Millennium Cohort Study (child age 11)				Fragile Families Study (child age 9)			
Mediator(s)	Indirect effect	SE	*p*	Mediator(s)	Indirect effect	SE	*p*
Coparenting only	**-0.010**	**0.004**	**0.016**	Coparenting only	**-0.048**	**0.016**	**0.003**
Coparenting and mother-child closeness	**-0.001**	**0.000**	**0.037**	Coparenting and mother's involvement	0.001	0.001	0.390
Coparenting and mother-child conflict	**-0.005**	**0.001**	**<.001**	Coparenting and mother's harsh discipline	**-0.003**	**0.002**	**0.044**
Coparenting and father-child closeness	0.000	0.000	0.118	Coparenting and father's involvement	0.000	0.002	0.970
Coparenting and father-child conflict	0.000	0.001	0.659	Coparenting and father's harsh discipline	0.000	0.001	0.911
Mother-child closeness only	-0.002	0.001	0.090	Mother's involvement only	0.006	0.006	0.340
Mother-child conflict only	**-0.008**	**0.002**	**<.001**	Mother's harsh discipline only	**-0.013**	**0.005**	**0.013**
Father-child closeness only	0.001	0.001	0.196	Father's involvement only	0.000	0.005	0.984
Father-child conflict only	-0.001	0.002	0.660	Father's harsh discipline only	0.000	0.003	0.908

Note: Models adjusted for parent-reported externalizing problems at age 3, parents' ages, ethnicity, education, marital status, number of children, children living elsewhere, resident grandparent and household income (MCS equivalised, FFS income to poverty ratio). Indirect effects are standardized with respect to predictors only. SE = standard error.

Figure A Path model of associations between couple supportiveness in infancy and middle childhood externalizing problems, allowing for prior levels of mediators, Fragile Families Study

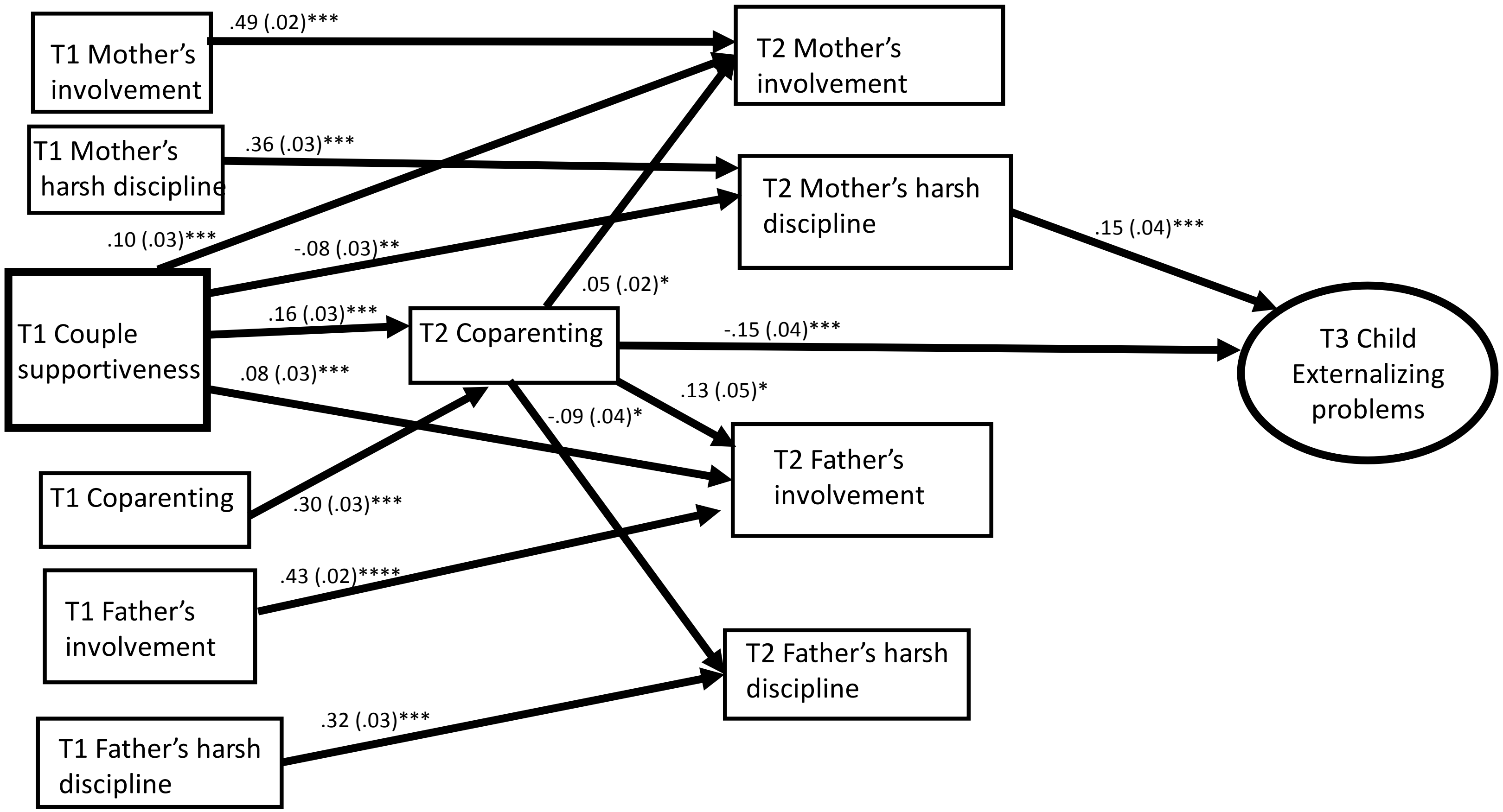

Note: T1 = age 1, T2 = age 3, T3 = age 9. Models adjusted for parents' age, ethnicity, education, marital status, number of children, non-resident children, resident grandparent and household income. For simplicity, significant correlations between T1 measures and between T2 measures, and all non-significant associations between constructs shown have been omitted. Figures show standardized coefficients with standard errors in parentheses. * denotes p <0.05, **p <0.01, ***p <0.001

Table G Indirect effects from couple supportiveness to age 9 externalising problems, allowing for prior measurement of mediators, Fragile Families Study

**Mediator(s)**	Indirect effect	SE	*p*
Coparenting only	**-0.026**	**0.008**	**0.002**
Coparenting and mother's involvement	0.000	0.000	0.904
Coparenting and mother's harsh discipline	-0.001	0.001	0.132
Coparenting and father's involvement	0.001	0.001	0.433
Coparenting and father's harsh discipline	0.000	0.001	0.634
Mother's involvement only	0.000	0.004	0.903
Mother's harsh discipline only	**-0.012**	**0.006**	**0.032**
Father's involvement only	0.003	0.004	0.401
Father's harsh discipline only	-0.001	0.003	0.651

Note: these effects are derived from the path model shown in Figure A above. Model adjusted for parents' age, ethnicity, education, marital status, number of children, non-resident children, resident grandparent and household income, coparenting and parenting at child age 1. Indirect effects standardized with respect to predictors only, with significant effects in bold typeface. SE = standard error.

Table H Indirect effects from couple supportiveness to children's externalising problems in middle childhood, according to source of information on partner supportiveness and coparenting

	Model A (using mother-reported partner supportiveness and coparenting)			Model B (using father-reported partner supportiveness and coparenting)			Comparison of Models A and B	
**Millennium Cohort Study**	Indirect effect	SE	*p*	Indirect effect	SE	*p*	z	*p*
All via coparenting (+/-parenting)	-0.045	0.005	<.001	-0.027	0.005	<.001	-2.546	0.011
All via parenting only	-0.061	0.006	<.001	-0.044	0.007	<.001	-1.844	0.065
Combined indirect pathways	-0.106	0.008	<.001	-0.071	0.009	<.001	-2.907	0.004
**Fragile Families Study**	Indirect effect	SE	*p*	Indirect effect	SE	*p*	z	*p*
All via coparenting (+/-parenting)	-0.029	0.012	0.015	-0.051	0.015	<.001	-1.145	0.252
All via parenting only	-0.013	0.007	0.079	-0.013	0.008	0.111	0.000	1.000
Combined indirect pathways	-0.042	0.014	0.002	-0.064	0.016	<.001	-1.035	0.301

Note: Models adjusted for parents' age, ethnicity, education, marital status, number of children, non-resident children, resident grandparent and household income. Indirect effects are standardized with respect to predictors only. SE = standard error.

Table I Comparison of indirect effects using alternative parenting measures, Millennium Cohort Study

	Model A (as Table 2, main text)			Model B (alternative parenting measures)			Model A vs B comparison	
	indirect effect	SE	*p*	indirect effect	SE	*p*	z	*p*
Via positive parenting	-0.019	0.004	<.001	-0.006	0.003	0.034	-2.600	0.009
Via negative parenting	-0.060	0.006	<.001	-0.040	0.005	<.001	-2.561	0.010

Note: In Model A positive parenting was measured using mother-child closeness, and father-child closeness; and negative parenting was measured using mother-child conflict, and father-child conflict. In Model B, positive parenting was measured using mother's involvement, and father's involvement; while negative parenting was measured using mother's harsh discipline and father-child conflict (harsh discipline was not available for fathers). Models adjusted for parents' age, ethnicity, education, marital status, number of children, non-resident children, resident grandparent and household income. Indirect effects are standardized with respect to predictors only. SE = standard error.
